# ConsisTNet: a spatio-temporal approach for consistent anatomical localization in endoscopic pituitary surgery

**DOI:** 10.1007/s11548-025-03369-2

**Published:** 2025-04-29

**Authors:** Zhehua Mao, Adrito Das, Danyal Z. Khan, Simon C. Williams, John G. Hanrahan, Danail Stoyanov, Hani J. Marcus, Sophia Bano

**Affiliations:** 1 Department of Computer Science, University College London, London, UK; 2 UCL Hawkes Institute, University College London, London, UK; 3 Department of Neurosurgery, National Hospital for Neurology and Neurosurgery, London, UK

**Keywords:** Endoscopic pituitary surgery, Temporal consistency, Anatomical localization, Volatility reduction, Real-time surgical guidance

## Abstract

****Purpose**:**

Automated localization of critical anatomical structures in endoscopic pituitary surgery is crucial for enhancing patient safety and surgical outcomes. While deep learning models have shown promise in this task, their predictions often suffer from frame-to-frame inconsistency. This study addresses this issue by proposing ConsisTNet, a novel spatio-temporal model designed to improve prediction stability.

****Methods**:**

ConsisTNet leverages spatio-temporal features extracted from consecutive frames to provide both temporally and spatially consistent predictions, addressing the limitations of single-frame approaches. We employ a semi-supervised strategy, utilizing ground-truth label tracking for pseudo-label generation through label propagation. Consistency is assessed by comparing predictions across consecutive frames using predicted label tracking. The model is optimized and accelerated using TensorRT for real-time intraoperative guidance.

****Results**:**

Compared to previous state-of-the-art models, ConsisTNet significantly improves prediction consistency across video frames while maintaining high accuracy in segmentation and landmark detection. Specifically, segmentation consistency is improved by 4.56 and 9.45% in IoU for the two segmentation regions, and landmark detection consistency is enhanced with a 43.86% reduction in mean distance error. The accelerated model achieves an inference speed of 202 frames per second (FPS) with 16-bit floating point (FP16) precision, enabling real-time intraoperative guidance.

****Conclusion**:**

ConsisTNet demonstrates significant improvements in spatio-temporal consistency of anatomical localization during endoscopic pituitary surgery, providing more stable and reliable real-time surgical assistance.

## Introduction

**Fig. 1:**
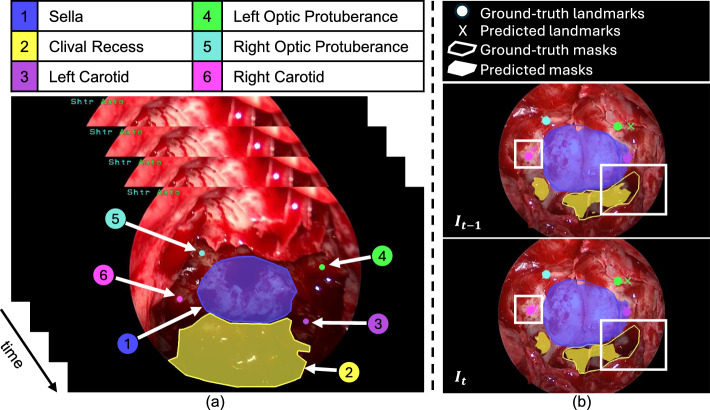
**a** Temporal sequence of critical anatomical structures during the sellar phase of eTSA. **b** An example of prediction inconsistency (from PitSurgRT [4]) between video frame $I_{t-1}$ and $I_{t}$, with inconsistent predictions highlighted by white boxes

The pituitary gland, located at the base of the brain near critical structures such as the optic nerves and carotid arteries, plays a vital role in hormone regulation [1]. Tumors in this gland can disrupt hormone secretion and impair vision by compressing the optic nerves [1]. For symptomatic patients, transsphenoidal surgery is the standard treatment, with the endoscopic transsphenoidal approach (eTSA) increasingly preferred due to its minimally invasive nature. This technique allows tumor removal through the nasal cavity and sphenoid sinus while minimizing damage to surrounding structures. However, accurately identifying safe entry points on the sphenoid bone, particularly in the sella region, remains challenging due to the lack of distinct anatomical features and the tumor’s invisibility (Fig. 1a). Errors during surgery can lead to severe complications, such as vision loss or carotid artery injury [1].

To enhance spatial orientation, optical tracking systems integrated with preoperative 3D imaging are commonly used in eTSA [2]. However, these systems disrupt surgical workflow, requiring surgeons to mentally retain localization information, which increases cognitive load. Additionally, preoperative imaging does not account for intraoperative anatomical shifts, further complicating decision making. These limitations underscore the need for real-time, intraoperative guidance systems that integrate seamlessly into the surgical process.

Recent advancements in computer vision have explored endoscopic video-based techniques to identify the sella and other critical anatomical structures. PAINet [3] was the first to address this, with PitSurgRT [4] improving accuracy and achieving real-time performance. However, these models rely on discrete image frames, ignoring the temporal continuity of video data. This can lead to inconsistent predictions across consecutive frames (Fig. 1b) [5], potentially confusing surgeons and increasing intraoperative risks [6].

To address these challenges, we propose **ConsisTNet**, a spatio-temporal model designed to ensure consistent anatomical localization during eTSA. ConsisTNet leverages features from consecutive video frames and employs a semi-supervised approach to enforce both spatial and temporal consistency, while based on HRNet [7] and ConvLSTM [8], our model introduces a novel pseudo-label generation method tailored for video data. This method enables the use of temporal information from video sequences, reducing prediction volatility and improving consistency. Unlike prior research [3, 4], which has largely overlooked this issue, our work focuses on enhancing the stability of landmark detection and segmentation in pituitary surgery while maintaining the high accuracy of PitSurgRT. The key contributions of this work are as follows:

1. A novel network architecture, **ConsisTNet**, that integrates spatio-temporal information to reduce prediction volatility during eTSA.
2. A pseudo-label generation method based on CoTracker2 [9] for temporal learning and consistency evaluation in the absence of ground-truth data.
3. A detailed analysis of the impact of temporal learning on reducing prediction variability in pituitary surgical video sequences.
4. A real-time implementation of **ConsisTNet** to meet the performance requirements of intraoperative guidance.

## Related works

Recent deep learning advances have substantially improved computer-assisted interventions [10–12]. For instance, Staartjes et al.  [11] employed a U-Net architecture to segment three endonasal anatomical structures in a proof-of-concept eTSA study. Das et al.  [12] integrated segmentation and object detection for instrument tracking, subsequently applying this approach for skill assessment in eTSA on a high-fidelity phantom. To jointly identify safe entry zones and surrounding critical structures in eTSA, Das et al.  [3] developed PAINet, a UNet++-based model for segmentation and landmark detection. Although PAINet showed promise, it faced challenges in real-time performance and detection accuracy. Mao et al. [4] addressed these limitations by introducing PitSurgRT, which integrates HRNet [7] to improve segmentation and landmark detection while achieving real-time inference. However, PitSurgRT processes video frames independently, ignoring the temporal continuity of video data and causing prediction inconsistencies (Fig. 1b).

To improve prediction consistency, researchers have incorporated temporal information into their models. Zhao et al. [13] proposed SSTAN, a semi-supervised spatio-temporal attention network that fuses a vision transformer-based attention mechanism with U-Net to segment polyps in video. Wang et al. [14] enhanced consistency by aggregating spatio-temporal features through ConvLSTM [8]. Furthermore, the multi-stage temporal convolutional network (MSTCN) [15] has been successfully employed to capture temporal patterns in video data.

Point-tracking methods have also leveraged temporal information. Teed and Deng [16] developed RAFT, which refines dense correspondences iteratively for accurate optical flow estimation. Doersch et al. [17] presented TAPIR, a method that tracks arbitrary points through per-frame initialization and temporal refinement, effectively handling occlusions and large displacements. Recently, Karaev et al. [9] introduced CoTracker2, a transformer-based model for multi-point tracking capable of preserving spatial relationships across frames, even under occlusions.

Building on these advancements, ConsisTNet combines spatio-temporal learning with semi-supervised techniques to improve real-time prediction stability in eTSA, specifically addressing the temporal consistency issues observed in previous works.

**Fig. 2:**
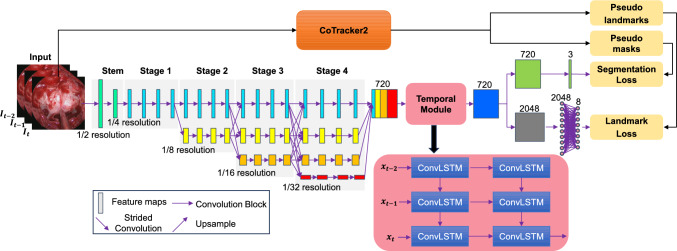
ConsisTNet architecture includes: a HRNet backbone; a temporal module; and dual heads. CoTracker2-generated pseudo-labels are utilized during training to enhance spatio-temporal learning

## Method

### Overview of ConsisTNet

As illustrated in Fig. 2, the proposed ConsisTNet combines HRNet for extracting high-resolution spatial features, ConvLSTM for capturing temporal features, and dual heads to produce temporally consistent segmentation masks and landmark coordinates, localizing the safe entry zone and critical anatomical structures, respectively. To enhance temporal granularity, CoTracker2-generated pseudo-labels are incorporated during training for loss calculations, propagating real labels to adjacent frames, and addressing the challenge of limited labeled video data. These strategies improve temporal consistency while preserving the high accuracy of PitSurgRT.

During training, each input sample comprises three consecutive frames, with real labels provided for the last frame. Two branches process the input sample: One uses CoTracker2 to generate pseudo-labels for the first two frames, while the other passes the sample through ConsisTNet to generate logits for loss computation. In inference, only ConsisTNet is used, predicting all three frames of each sample simultaneously.

### HRNet as the spatial module

HRNet’s ability to maintain both high- and low-resolution representations [7] has proven highly effective in previous work [4] for eTSA images, outperforming networks such as UNet++, DeepLabv3+, and PSPNet [3]. ConsisTNet employs HRNet as its backbone. The input image is downsampled to 1/4 of its original size via two $3 \times 3$ convolutional layers with a stride of 2. HRNet consists of four stages, each with four parallel branches that maintain feature maps at resolutions of 1/4, 1/8, 1/16, and 1/32. The first stage includes 4 residual units, while subsequent stages add branches, doubling feature map width at each stage transition. By the final stage, the feature map widths are 48, 96, 192, and 384 channels. High- and low-resolution feature maps are fused at each stage, ensuring effective multi-scale information exchange. After the final stage, low-resolution feature maps are upsampled and fused with high-resolution ones, resulting in a final high-resolution feature map with 720 channels.

### Temporal module

ConvLSTM is integrated into ConsisTNet to capture temporal features in eTSA videos while preserving spatial details, making it well-suited for both segmentation and landmark detection tasks. The temporal module operates on HRNet-extracted feature maps using two ConvLSTM layers, each with 720 input and hidden channels, preserving high-resolution features for accurate localization. Both layers use a $3 \times 3$ kernel and a stride of 1 to capture spatial details while learning temporal dependencies. The two-layer module processes sequences of feature maps, updating hidden and cell states to learn short- and long-term dependencies, capturing hierarchical temporal features.

ConvLSTM cells employ four gates—input, forget, output, and cell state—to control information flow, selectively retaining or discarding data to maintain relevant features [8]. This enhances prediction consistency, reducing fluctuations between frames and leading to smoother, more stable predictions, especially during gradual surgical view changes. ConvLSTM’s ability to preserve spatial–temporal coherence while maintaining real-time performance makes it ideal for improving ConsisTNet’s stability.

### Dual-head outputs

Following the temporal module, two heads are connected: one for segmenting the sella and clival recess and another for detecting landmarks. The segmentation head uses a $1 \times 1$ convolutional layer with stride 1, followed by batch normalization, ReLU activation, and a final $1 \times 1$ convolutional layer with argmax activation and upsampling to recover the original resolution. For landmark detection, feature maps pass through a $1 \times 1$ convolutional layer with stride 1, average pooling, ReLU activation, and fully connected layers to output the coordinates of four landmarks.

**Fig. 3:**
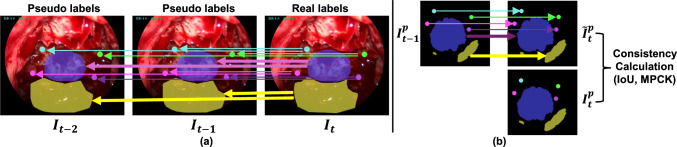
CoTracker2 is utilized for: **a** pseudo-label generation at frames $I_{t-1}$ and $I_{t-2}$ and **b** consistency evaluation. The arrows indicate the propagation of annotations in **a** and predictions in **b** across consecutive frames using CoTracker2

### Tracking-based pseudo-label generation

Manual video annotation is often sparse due to the high workload, typically at lower frame rates, which disrupts temporal coherence between frames and limits the network’s ability to learn frame-to-frame consistency. To mitigate this, we use CoTracker2 to generate pseudo-labels for unannotated frames, enhancing temporal continuity in the training data. CoTracker2 can handle occlusions caused by surgical instruments in eTSA, making it ideal for this task. As shown in Fig. 3a, CoTracker2 can propagate real labels (landmarks and segmentation masks) from the annotated frame to unannotated frames to generate pseudo-labels (e.g., from $I_t$ to $I_{t-1}$ and $I_{t-2}$ in Fig. 3a). Since CoTracker2 was originally developed for point tracking, for mask propagation, we sample them into sparse feature points, track them across frames, and reconstruct the mask using concave hull geometry. This method ensures accurate pseudo-labels for both landmarks and masks, maintaining temporal coherence.

### Loss function

In our previous work [4], we demonstrated the effectiveness of using Dice loss for segmentation, as well as Wing loss and Focal loss (FL) for landmark detection. In this paper, we extend the loss function by incorporating PyTorch’s smooth L1 loss for both tasks. This extension is motivated by the minimal movement observed during the sellar phase of surgery, where the endoscope remains largely stationary. As a result, predictions for segmentation and landmark detection should exhibit temporal consistency across frames. Unlike Dice, Wing, and Focal losses, which measure differences between predictions and ground-truth/pseudo-labels, smooth L1 loss promotes temporal coherence by computing differences between predictions of consecutive frames. This ensures consistency over time. The overall loss function is defined as:

1 $$\begin{aligned} \textrm{Loss}= &   \big (w_{\textrm{1}} \cdot \textrm{Dice} + w_{\textrm{2}} \cdot \textrm{L1}_{\textrm{seg}}\big ) \nonumber \\  &   + \big (w_{\textrm{3}} \cdot \textrm{Wing} + w_{\textrm{4}} \cdot \textrm{FL} + w_{\textrm{5}} \cdot \textrm{L1}_{\textrm{ldmk}}\big ), \end{aligned}$$

where $\textrm{L1}_{\textrm{seg}}$ and $\textrm{L1}_{\textrm{ldmk}}$ are smooth L1 losses for the segmentation and landmark detection tasks, respectively. The weights $w_{1}$, $w_{2}$, $w_{3}$, $w_{4}$, and $w_{5}$ are hyperparameters that balance the contributions of the individual loss components.

## Experimental setup

### Dataset

The dataset consists of 635 manually annotated frames, created by four neurosurgeons, extracted from 64 complete eTSA videos. The videos have a mean duration of 74 min, and each video is divided into four distinct surgical phases [4]. Our work focuses on the sellar phase, which is critical for identifying key anatomical structures. For each video, a 10-second segment preceding sellotomy was manually annotated, with frames initially sampled at 1 FPS. Additional details about the dataset setup can be found in our previous works [3, 4]. Due to the inherent difficulty of labeling invisible features on sphenoidal bones, some anatomical structures remain partially annotated. The sella and clival recess are annotated in all frames, while other structures are labeled in up to 65% of the frames. Our primary focus is on the sella region, where the tumor is located posteriorly. While the clival recess is typically visible during the sellar phase, it is clinically less significant. Nevertheless, it can serve as a reference to help the model identify the locations of other anatomies.

To address the challenge of sparse annotations and to enable the model to learn spatio-temporal continuity, we resampled the 10-second clips at 3 FPS for training. This frame rate balances temporal continuity and computational efficiency. The manually annotated frames serve as ground truth, while pseudo-labels are generated for the additional frames using tracking methods, as detailed in Sect. Tracking-based pseudo-label generation. This process augments the dataset with 1,270 pseudo-labeled frames, increasing the total number of annotated frames to 1,905. For evaluation, we adopt a fivefold cross-validation strategy consistent with prior works [4], ensuring that all frames from the same patient are assigned to the same fold. During inference, the same 3 FPS sampling rate is applied to maintain consistency with the training setup.

### Evaluation metric

Given the relatively small size of our dataset (64 surgical videos), to maximize the data available for training while maintaining rigorous validation, we evaluate the model in two aspects by using fivefold cross-validation [18]: prediction performance and consistency.

**Performance evaluation**: Performance is evaluated by comparing predictions ${I}_{t}^p$ with ground truth $I_{t}^g$ at frame ${I}_{t}$. Metrics include Intersection over Union (IoU) and ${\hbox {F}_{1}\hbox {-score}}$ score for segmentation, as well as mean distance error (mDistance) and the percentage of correct keypoints (MPCK5, MPCK10, MPCK20) for landmarks. MPCK10, for instance, denotes the percentage of landmarks within 10% of the image height (72 pixels) from the ground truth. Our prior clinical study [4] validated MPCK20 as a reliable and clinically meaningful criterion for selecting models that are sufficiently accurate for surgical guidance.

**Table 1:** Performance and consistency evaluation (mean ± std, fivefold cross-validation)

Method	Sella	Clival recess	Sella	Clival recess	Landmarks			
	IoU (%) $\uparrow$		${\hbox {F}_{1}\hbox {-score}}$ (%) $\uparrow$		MPCK5 (%) $\uparrow$	MPCK10 (%) $\uparrow$	MPCK20 (%) $\uparrow$	mDistance (pixel) $\downarrow$
	Performance evaluation							
PitSurgRT [4]	$66.59\pm 2.29$	$\varvec{45.81\pm 7.19}$	$79.92\pm 1.66$	$\varvec{62.51\pm 6.70}$	${26.08\pm 7.67}$	$68.88\pm 8.55$	$94.75\pm 4.79$	${63.05\pm 10.20}$
PAINet [3]	$64.31\pm 2.85$	$45.32\pm 9.17$	$78.24\pm 2.11$	$61.79\pm 9.28$	$7.92\pm 1.71$	$34.38\pm 7.47$	$83.31\pm 6.83$	$97.12\pm 11.06$
MSTCN [15]	$64.63\pm 4.47$	$41.73\pm 8.61$	$78.43\pm 3.28$	$58.36\pm 8.80$	$13.72\pm 11.27$	$41.05\pm 13.85$	$85.39\pm 7.84$	$89.76\pm 18.38$
SSTAN [13]	$41.52\pm 3.67$	$23.69\pm 4.63$	$58.58\pm 3.61$	$38.07\pm 6.35$	$13.96\pm 6.43$	$43.49\pm 13.78$	$86.24\pm 9.82$	$88.58\pm 20.20$
ConsisTNet	$\varvec{66.84\pm 3.31}$	$45.08\pm 7.43$	$\varvec{80.62\pm 2.29}$	$60.24\pm 8.21$	$\varvec{34.05\pm 6.54}$	$\varvec{74.74\pm 8.97}$	$\varvec{96.34\pm 4.94}$	$\varvec{55.10\pm 9.77}$
	Consistency Evaluation							
PitSurgRT [4]	$82.85\pm 1.13$	$67.41\pm 4.87$	$90.62\pm 0.68$	$80.43\pm 3.58$	$94.14\pm 2.68$	$98.69\pm 1.01$	$99.93\pm 0.13$	$12.79\pm 2.47$
PAINet [3]	$81.29\pm 1.83$	$68.23\pm 2.90$	$89.67\pm 1.12$	$81.08\pm 2.03$	$82.70\pm 2.64$	$97.85\pm 0.69$	$99.96\pm 0.05$	$23.49\pm 1.53$
MSTCN [15]	$80.72\pm 2.51$	$66.83\pm 5.46$	$89.31\pm 1.55$	$79.98\pm 4.10$	$\varvec{97.92\pm 0.53}$	$99.54\pm 0.30$	$99.98\pm 0.04$	$4.28\pm 0.53$
SSTAN [13]	$62.71\pm 4.39$	$40.71\pm 3.57$	$76.99\pm 3.27$	$57.77\pm 3.61$	$98.09\pm 0.48$	$99.49\pm 0.31$	$99.93\pm 0.05$	$\varvec{4.24\pm 0.60}$
ConsisTNet	$\varvec{86.63\pm 1.68}$	$\varvec{73.78\pm 3.38}$	$\varvec{92.83\pm 0.97}$	$\varvec{84.87\pm 2.24}$	$97.92\pm 2.02$	$\varvec{99.70\pm 0.18}$	$\varvec{99.98\pm 0.04}$	$7.18\pm 1.75$

Bolded values represent the highest performance for each metric in the respective column

**Consistency evaluation**: Inspired by Varghese et al.  [19], temporal consistency is evaluated using CoTracker2. As shown in Fig. 3b, the model’s segmentation and landmark predictions ${I}_{t-1}^p$ are propagated from frame ${I}_{t-1}$ to its consecutive frame ${I}_{t}$ using CoTracker2, resulting in a tracked prediction $\tilde{I}_{t}^p$. Comparing $\tilde{I}_{t}^p$ with model predictions ${I}_{t}^p$ at frame ${I}_{t}$, we calculate the model’s temporal consistency. Metrics such as IoU, ${\hbox {F}_{1}\hbox {-score}}$, mDistance, and MPCK are used for this evaluation.

### Training details

Training and validation are performed on a workstation with an NVIDIA RTX A6000 GPU (48 GB). The HRNet backbone, using pre-trained weights from PitSurgRT [4], remains frozen while training other components of ConsisTNet. Optimization uses SGD with a 0.9 momentum, and the learning rate starts at 0.01, decaying linearly to 0.0001 over 200 epochs. For the first 50 epochs, only the temporal module and segmentation head are trained, with the landmark detection head frozen. In the remaining 150 epochs, all components except for HRNet are trained jointly. The weights for $w_{1}$, $w_{2}$, $w_{3}$, $w_{4}$, and $w_{5}$ are set to 0.9, 0.1, 0.8, 0.2, and 0.005, respectively.

The proposed method is implemented in PyTorch 1.13.1, using Python 3.8.18 and CUDA 11.5. The code is available at https://github.com/ZH-Mao/PitVideo.git. To achieve real-time performance, we optimize ConsisTNet on NVIDIA GPUs using the TensorRT^1^ technique with 32-bit floating point (FP32) and FP16.

## Results and discussion

### Comparison with state-of-the-art methods

**Quantitative analysis:** We evaluate the performance of the proposed ConsisTNet against two image-based models, PitSurgRT [4] and PAINet [3], as well as two video-based models, MSTCN [15] and SSTAN [13]. All models, except PAINet, use the HRNet backbone with identical weights; PAINet is based on a pretrained EfficientNetB3 backbone [3]. While PitSurgRT and PAINet are trained solely with real labels, the video-based models (MSTCN, SSTAN, and ConsisTNet) utilize both real and pseudo-labels. The comparative results based on fivefold cross-validation are presented in Table 1.

As shown in the upper portion of Table 1, our proposed ConsisTNet achieves segmentation and landmark detection accuracies comparable to those of PitSurgRT. Moreover, it outperforms other image-based and video-based methods, including PAINet, MSTCN, and SSTAN, in both accuracy and consistency metrics. Regarding the consistency evaluation, the lower part of Table 1 illustrates a significant improvement in prediction stability across consecutive video frames. Specifically, ConsisTNet increases segmentation consistency by 4.56% for the sella and 9.45% for the clival recess compared to PitSurgRT in IoU. In terms of landmark detection, ConsisTNet markedly enhances consistency, reducing the mean distance error from $12.79 \pm 2.47$ pixels to $7.18 \pm 1.75$ pixels—a relative improvement of 43.86%.

**Fig. 4:**
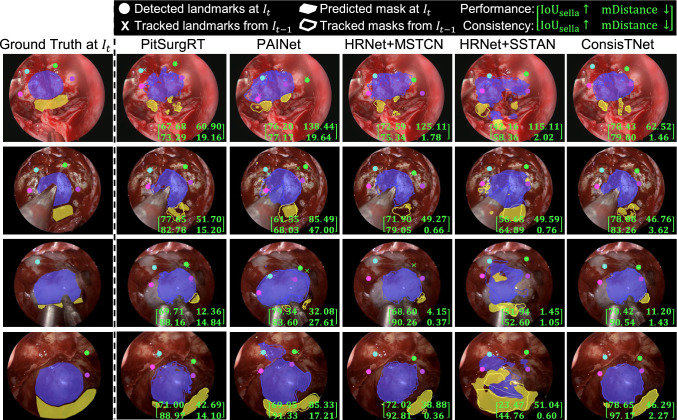
Qualitative comparison of prediction performance and consistency between the proposed and compared methods. The IoU for the focused area, sella, is displayed in the bottom right of each image

**Table 2:** Ablation study for assessing the accuracy of the CoTracker2 compared with RAFT and TAPIR (single fold)

Method	Performance evaluation							
	Sella	Clival recess	Sella	Clival recess	Landmarks			
	IoU (%) $\uparrow$		${\hbox {F}_{1}\hbox {-score}}$ (%) $\uparrow$		MPCK5 (%) $\uparrow$	MPCK10 (%) $\uparrow$	MPCK20 (%) $\uparrow$	mDistance (pixel) $\downarrow$
RAFT [16]	86.59	70.09	92.81	82.41	96.38	99.26	99.59	6.93
TAPIR [17]	55.46	34.66	71.34	51.47	23.37	93.03	100.00	46.01
CoTracker2 [9]	$\varvec{89.23}$	$\varvec{74.11}$	$\varvec{94.31}$	$\varvec{85.12}$	$\varvec{99.33}$	$\varvec{100.00}$	$\varvec{100.00}$	$\varvec{4.71}$

Bolded values represent the highest performance for each metric in the respective column

**Table 3:** Ablation study for assessing the effectiveness of the CoTracker2 module, temporal module, and consistency loss (single fold)

Method	Sella	Clival recess	Sella	Clival recess	Landmarks			
	IoU (%) $\uparrow$		${\hbox {F}_{1}\hbox {-score}}$ (%) $\uparrow$		MPCK5 (%) $\uparrow$	MPCK10 (%) $\uparrow$	MPCK20 (%) $\uparrow$	mDistance (pixel) $\downarrow$
	Performance Evaluation							
PitSurgRT	69.98	$\varvec{53.31}$	82.34	$\varvec{69.55}$	24.14	78.68	97.81	55.06
PitSurgRT+CoTracker2	63.22	45.75	77.47	62.78	14.73	61.44	96.55	66.87
PitSurgRT+Cotracker2+ConvLSTM	67.74	47.19	80.76	64.12	22.88	40.44	89.97	84.60
PitSurgRT+Cotracker2+ConvLSTM +Consistency loss	$\varvec{71.16}$	52.34	$\varvec{83.15}$	68.71	$\varvec{32.60}$	$\varvec{84.64}$	$\varvec{100.00}$	$\varvec{48.10}$
	Consistency Evaluation							
PitSurgRT	82.90	68.83	90.65	81.54	95.65	98.55	100.00	11.22
PitSurgRT+CoTracker2	80.83	64.68	89.40	78.55	94.86	98.46	100.00	12.89
PitSurgRT+Cotracker2+ConvLSTM	83.67	70.02	91.11	82.37	98.39	99.79	99.89	8.33
PitSurgRT+Cotracker2+ConvLSTM +Consistency loss	$\varvec{86.70}$	$\varvec{73.43}$	$\varvec{92.88}$	$\varvec{84.68}$	$\varvec{99.69}$	$\varvec{99.90}$	$\varvec{100.00}$	$\varvec{5.95}$

Bolded values represent the highest performance for each metric in the respective column

**Qualitative analysis:** Fig. 4 presents qualitative results from four surgeries, comparing prediction accuracy and consistency of segmentation and landmark detection across video frames. A matrix in the bottom right of each image provides quantitative results. ConsisTNet demonstrates similar prediction accuracy to PitSurgRT (first row of matrices), often achieving the best or comparable results in sella segmentation and landmark detection among competing methods. While MSTCN and SSTAN show strong landmark detection consistency with lower mDistance (second row of matrices), they struggle with prediction accuracy and segmentation consistency, e.g., they exhibit significant errors for landmark detection accuracy (over 100 pixels) in the first case and lower segmentation consistency for all four cases. Overall, ConsisTNet outperforms other methods by balancing superior consistency and good prediction accuracy.

Using TensorRT, we accelerated the trained models. Compared to the PyTorch model (inference speed: $10.13 \pm 0.03$ FPS), the accelerated models achieved speeds of $113.10 \pm 8.22$ FPS (FP32) and $202.00 \pm 7.09$ FPS (FP16), without performance loss.

### Ablation studies

**Accuracy of pseudo-label generation:** Following the pseudo-labeling approach described in Sect. Tracking-based pseudo-label generation, we evaluate the accuracy of CoTracker2 by comparing it with two state-of-the-art methods, RAFT [16] and TAPIR [17]. We propagate annotations from frames with ground-truth labels to other labeled frames and compare the resulting pseudo-labels against the real labels. For point tracking and pseudo-label generation using RAFT, we utilized its optical flow estimates to propagate points across frames. Specifically, (1) for each point in frame $I_t$, we computed the optical flow to frame $I_{t-1}$; (2) the resulting flow vectors were then used to estimate the new position of the point in frame $I_{t-1}$; (3) this process was iteratively repeated for subsequent frames. For the point-tracking method TAPIR, we followed the same pipeline used for CoTracker2 to generate pseudo-labels, as described in Sect. Tracking-based pseudo-label generation.

As shown in Table 2, CoTracker2 consistently outperforms RAFT and TAPIR for both landmarks and masks, demonstrating its robustness and confirming that it is the most suitable method for generating high-quality pseudo-labels in our occlusion-prone surgical videos.

**Effectiveness of each module in ConsisTNet:** We perform four ablation experiments to assess the effectiveness of the CoTracker2 module, temporal module, and consistency loss: (1) PitSurgRT trained with real labels only; (2) PitSurgRT trained with real and pseudo-labels; (3) PitSurgRT with ConvLSTM (no consistency loss), trained with real and pseudo-labels. (4) PitSurgRT with ConvLSTM and consistency loss, trained with real and pseudo-labels.

The results in Table 3 highlight contributions of each component. Introducing CoTracker2 alone does not boost accuracy or consistency because the sellar phase is relatively stationary and pseudo-labels—generated between consecutive ground-truth labels that are 1 s apart—offer limited additional variation. Moreover, if real labels are obstructed by surgical instruments, it can lead to incomplete or inaccurate pseudo-labels. However, adding the temporal module (ConvLSTM) mitigates these issues by modeling frame-to-frame dependencies. When combined with the consistency loss, our model matches the baseline’s accuracy while significantly improving consistency (see Table 3). Specifically, the IoU consistency improves by 4.58% (sella) and 6.68% (clival recess), and landmark detection consistency improves by 46.97%, reducing the mean distance error from 11.22 pixels to 5.95 pixels. A fivefold cross-validation in Table 1 further confirms the robustness of these enhancements.

**Table 4:** Ablation study for assessing the impact of the hyperparameters on model performance (single fold)

Weights $[w_1, w_2, w_3, w_4, w_5]$	Sella	Clival recess	Landmarks	Sella	Clival recess	Landmarks	Overall* $\uparrow$
	IoU (%) $\uparrow$		MPCK10 (%) $\uparrow$	IoU (%) $\uparrow$		MPCK10 (%) $\uparrow$	
	Performance Evaluation			Consistency Evaluation			
[0.9, 0.00, 0.8, 0.2, 0.000]	67.74	47.19	40.44	83.67	70.02	99.79	274.54
[0.9, 0.01, 0.8, 0.2, 0.001]	71.29	56.73	69.28	85.53	70.87	99.46	310.95
[0.9, 0.05, 0.8, 0.2, 0.001]	70.85	55.57	78.37	85.57	72.31	100.00	320.52
[0.9, 0.10, 0.8, 0.2, 0.005]	71.16	52.34	84.64	86.70	73.43	99.90	$\varvec{326.36}$
[0.9, 0.15, 0.8, 0.2, 0.010]	70.90	53.39	80.56	86.57	74.62	99.79	323.10
[0.9, 0.20, 0.8, 0.2, 0.020]	70.81	52.69	80.13	86.96	74.89	100.00	322.81

Note: * The overall score is calculated as $\mathrm {\left[ \frac{IoU_{S} + IoU_{C}}{2} + MPCK10\right] _{Per.} + \left[ \frac{IoU_{S} + IoU_{C}}{2} + MPCK10\right] _{Con.}}$, where $\mathrm {IoU_{S}}$ and $\mathrm {IoU_{C}}$ represent the IoU metrics for the Sella and Clival recess, respectively. Here, $\mathrm {Per.}$ and $\mathrm {Con.}$ are abbreviations for performance and consistency evaluations, respectively. The bolded value represents the overall highest performance and consistency achieved by the model

Interestingly, adding CoTracker2 alone can initially degrade performance due to noisy pseudo-labels, especially when the real labels are occluded by instruments. Although introducing ConvLSTM recovers some performance, it still lags behind the baseline, suggesting that temporal modeling alone is insufficient for fully leveraging pseudo-labels. The consistency loss proves crucial for refining these labels and temporal features. Only with all components—CoTracker2, ConvLSTM, and the consistency loss—does performance exceed the baseline, demonstrating a synergistic effect for surgical video analysis.

**Selection of hyperparameters:** Following PitSurgRT [4], we incorporate a temporal module and a pseudo-label generation module to capture temporal continuity in this work. Alongside the Dice, Wing, and Focal losses from PitSurgRT [4], we add two L1 losses to foster frame-to-frame prediction consistency. While we retain the same weights for Dice, Wing, and Focal losses, we fine-tune the L1 loss weights. Table 4 illustrates the impact of these hyperparameters on model performance. To balance accuracy and consistency, we consider an overall score (last column in Table 4), which indicates that assigning weights $w_{1}=0.9,\, w_{2}=0.1,\, w_{3}=0.8,\, w_{4}=0.2,$ and $w_{5}=0.005$ is optimal for this task.

## Conclusion

This paper introduced ConsisTNet, a novel spatio-temporal model designed to enhance consistency in anatomical localization during eTSA. ConsisTNet integrates HRNet for feature extraction, a temporal module using ConvLSTM, and CoTracker2 for pseudo-label generation and consistency evaluation. Our results demonstrate that ConsisTNet outperforms both image-based and video-based state-of-the-art methods in accuracy and consistency. Notably, it achieved significant improvements in segmentation and landmark detection consistency compared to our previous model, PitSurgRT. The optimized implementation using TensorRT enables real-time performance. This work represents a significant step toward more reliable and stable anatomical localization in eTSA, potentially enhancing surgical precision and safety. Future work will deploy this system and assess clinical impact. In addition, given the relatively small size of our current dataset, we are actively working on collecting and annotating additional surgical videos, which will enable more comprehensive validation of our approach in future studies.

## Footnotes

1 https://developer.nvidia.com/tensorrt.

## References

[1] Cavallo LM, Somma T, Solari D, Iannuzzo G, Frio F, Baiano C, Cappabianca P (2019) Endoscopic endonasal transsphenoidal surgery: history and evolution. World Neurosurg 127:686–694 31266131 10.1016/j.wneu.2019.03.048

[2] Enkaoua A, Islam M, Ramalhinho J, Dowrick T, Booker J, Khan DZ, Marcus HJ, Clarkson MJ (2023) Image-guidance in endoscopic pituitary surgery: an in-silico study of errors involved in tracker-based techniques. Front Surgery, 10 10.3389/fsurg.2023.1222859 PMC10540627 37780914

[3] Das A, Khan DZ, Williams SC, Hanrahan JG, Borg A, Dorward NL, Bano S, Marcus HJ, Stoyanov D (2023) A multi-task network for anatomy identification in endoscopic pituitary surgery. In: International conference on medical image computing and computer-assisted intervention, pp 472–482. Springer

[4] Mao Z, Das A, Islam M, Khan DZ, Williams SC, Hanrahan JG, Borg A, Dorward NL, Clarkson MJ, Stoyanov D, et al (2024) Pitsurgrt: real-time localization of critical anatomical structures in endoscopic pituitary surgery. Int J Comput Assist Radiol Surg, pp 1–8 10.1007/s11548-024-03094-2 PMC11178578 38528306

[5] Zhou T, Porikli F, Crandall DJ, Van Gool L, Wang W (2022) A survey on deep learning technique for video segmentation. IEEE Trans Pattern Anal Mach Intell 45(6):7099–7122 10.1109/TPAMI.2022.3225573 36449595

[6] Marcus HJ, Pratt P, Hughes-Hallett A, Cundy TP, Marcus AP, Yang G-Z, Darzi A, Nandi D (2015) Comparative effectiveness and safety of image guidance systems in neurosurgery: a preclinical randomized study. J Neurosurg 123(2):307–313 25909567 10.3171/2014.10.JNS141662 PMC4521142

[7] Wang J, Sun K, Cheng T, Jiang B, Deng C, Zhao Y, Liu D, Mu Y, Tan M, Wang X et al (2020) Deep high-resolution representation learning for visual recognition. IEEE Trans Pattern Anal Mach Intell 43(10):3349–3364 10.1109/TPAMI.2020.2983686 32248092

[8] Shi X, Chen Z, Wang H, Yeung D-Y, Wong W-K, Woo W-c (2015) Convolutional lstm network: A machine learning approach for precipitation nowcasting. Adv Neural Inf Process Syst, 28

[9] Karaev N, Rocco I, Graham B, Neverova N, Vedaldi A, Rupprecht C (2025) Cotracker: it is better to track together. In: European conference on computer vision, pp 18–35. Springer

[10] Madani A, Namazi B, Altieri MS, Hashimoto DA, Rivera AM, Pucher PH, Navarrete-Welton A, Sankaranarayanan G, Brunt LM, Okrainec A et al (2022) Artificial intelligence for intraoperative guidance: using semantic segmentation to identify surgical anatomy during laparoscopic cholecystectomy. Ann Surg 276(2):363–369 33196488 10.1097/SLA.0000000000004594 PMC8186165

[11] Staartjes VE, Volokitin A, Regli L, Konukoglu E, Serra C (2021) Machine vision for real-time intraoperative anatomic guidance: a proof-of-concept study in endoscopic pituitary surgery. Operat Neurosurg 21(4):242–247 10.1093/ons/opab187 34131753

[12] Das A, Sidiqi B, Mennillo L, Mao Z, Brudfors M, Xochicale M, Khan DZ, Newall N, Hanrahan JG, Clarkson MJ, et al (2024) Automated surgical skill assessment in endoscopic pituitary surgery using real-time instrument tracking on a high-fidelity bench-top phantom. Healthcare Technol Lett 10.1049/htl2.12101 PMC11665785 39720762

[13] Zhao X, Wu Z, Tan S, Fan D-J, Li Z, Wan X, Li G (2022) Semi-supervised spatial temporal attention network for video polyp segmentation. In: International conference on medical image computing and computer-assisted intervention, pp 456–466. Springer

[14] Wang J, Jin Y, Wang L, Cai S, Heng P-A, Qin J (2021) Efficient global-local memory for real-time instrument segmentation of robotic surgical video. In: Medical image computing and computer assisted intervention–MICCAI 2021: 24th international conference, Strasbourg, France, September 27–October 1, 2021, Proceedings, Part IV 24, pp 341–351. Springer

[15] Li S, Farha YA, Liu Y, Cheng M-M, Gall J (2020) Ms-tcn++: Multi-stage temporal convolutional network for action segmentation. IEEE Trans Pattern Anal Mach Intell 45(6):6647–6658 10.1109/TPAMI.2020.3021756 32886607

[16] Teed Z, Deng J (2020) Raft: Recurrent all-pairs field transforms for optical flow. In: Computer vision–ECCV 2020: 16th European conference, Glasgow, UK, August 23–28, 2020, Proceedings, Part II 16, pp 402–419. Springer

[17] Doersch C, Yang Y, Vecerik M, Gokay D, Gupta A, Aytar Y, Carreira J, Zisserman A (2023) Tapir: tracking any point with per-frame initialization and temporal refinement. In: Proceedings of the IEEE/CVF international conference on computer vision, pp 10061–10072

[18] Bradshaw TJ, Huemann Z, Hu J, Rahmim A (2023) A guide to cross-validation for artificial intelligence in medical imaging. Radiol Artif Intell 5(4):220232 10.1148/ryai.220232 PMC10388213 37529208

[19] Varghese S, Bayzidi Y, Bar A, Kapoor N, Lahiri S, Schneider JD, Schmidt NM, Schlicht P, Huger F, Fingscheidt T (2020) Unsupervised temporal consistency metric for video segmentation in highly-automated driving. In: Proceedings of the IEEE/CVF conference on computer vision and pattern recognition workshops, pp 336–337

